# Supplementary material for: DMRforPairs: identifying Differentially Methylated Regions between unique samples using array based methylation profiles
Source: BMC Bioinformatics. 2014 May 15;15:141. doi: 10.1186/1471-2105-15-141 (PMC4046028; doi:10.1186/1471-2105-15-141)
Supplement: Additional file 2 — DMRforPairs output for the comparison of A431-MCF7 and NA17018-NA17105. Please start from the HTML files in each folder. Available via the BMC Bioinformatics website. [file 1471-2105-15-141-S2.zip › 1394847754114233_MOESM2_ESM/NA17105M_NA17018F/figures/32635.pdf]

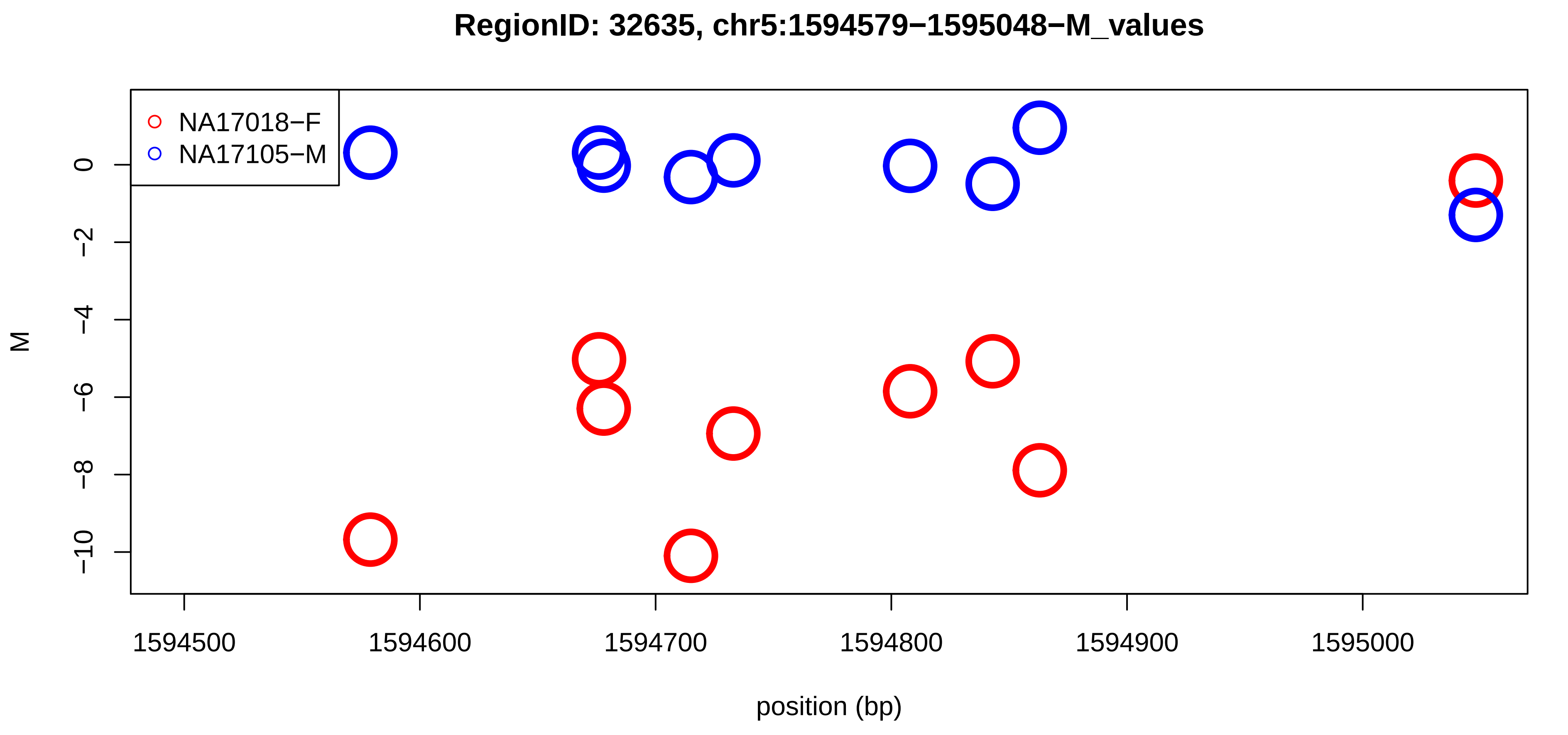
RegionID: 32635, chr5:1594579−1595048−M_values
NA17018−F
NA17105−M
M
0
−2
−4
−6
−8
−10
1594500
1594600
1594700
1594800
1594900
1595000
position (bp)

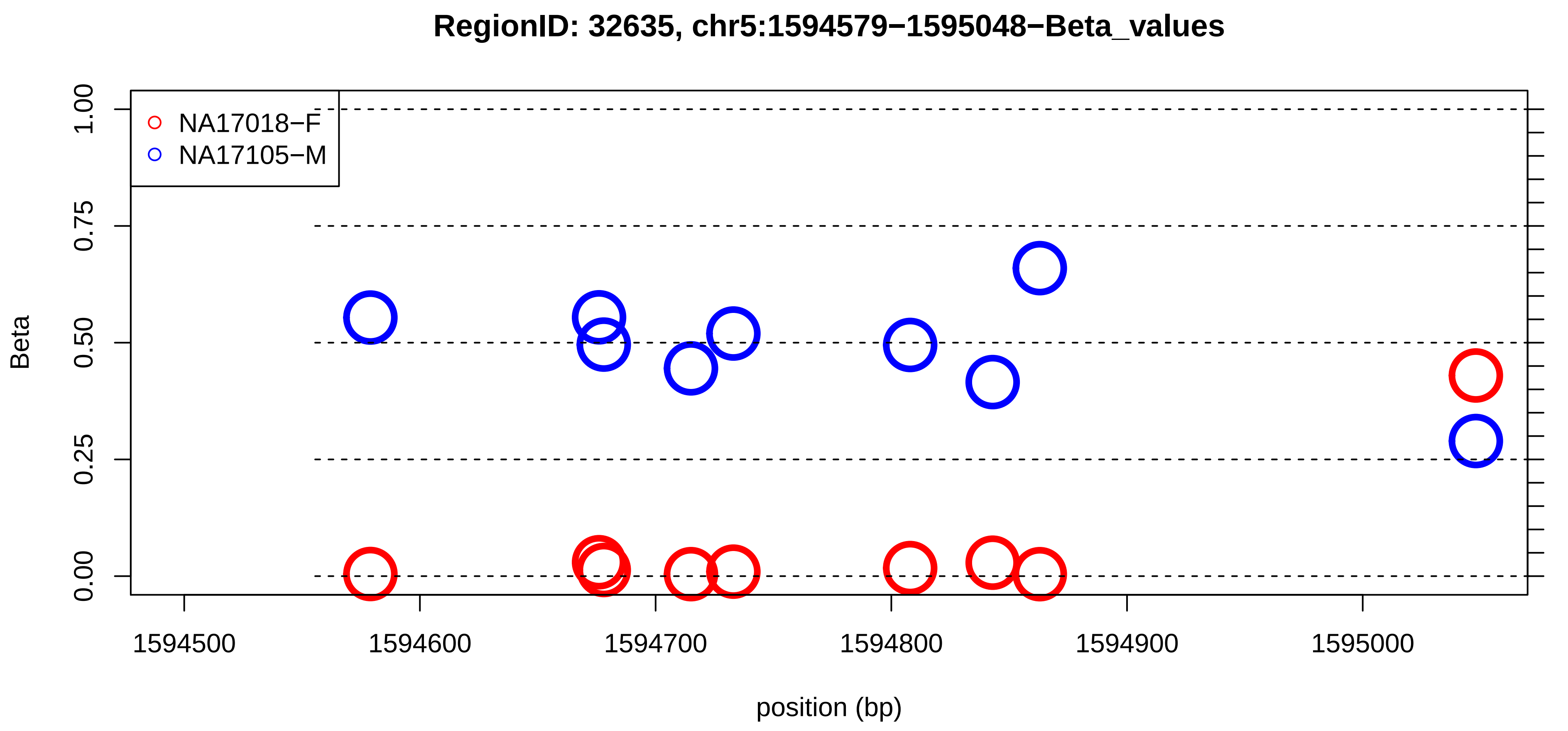
RegionID: 32635, chr5:1594579−1595048−Beta_values
NA17018−F
NA17105−M
Beta
0.00
0.25
0.50
0.75
1.00
1594500
1594600
1594700
1594800
1594900
1595000
position (bp)

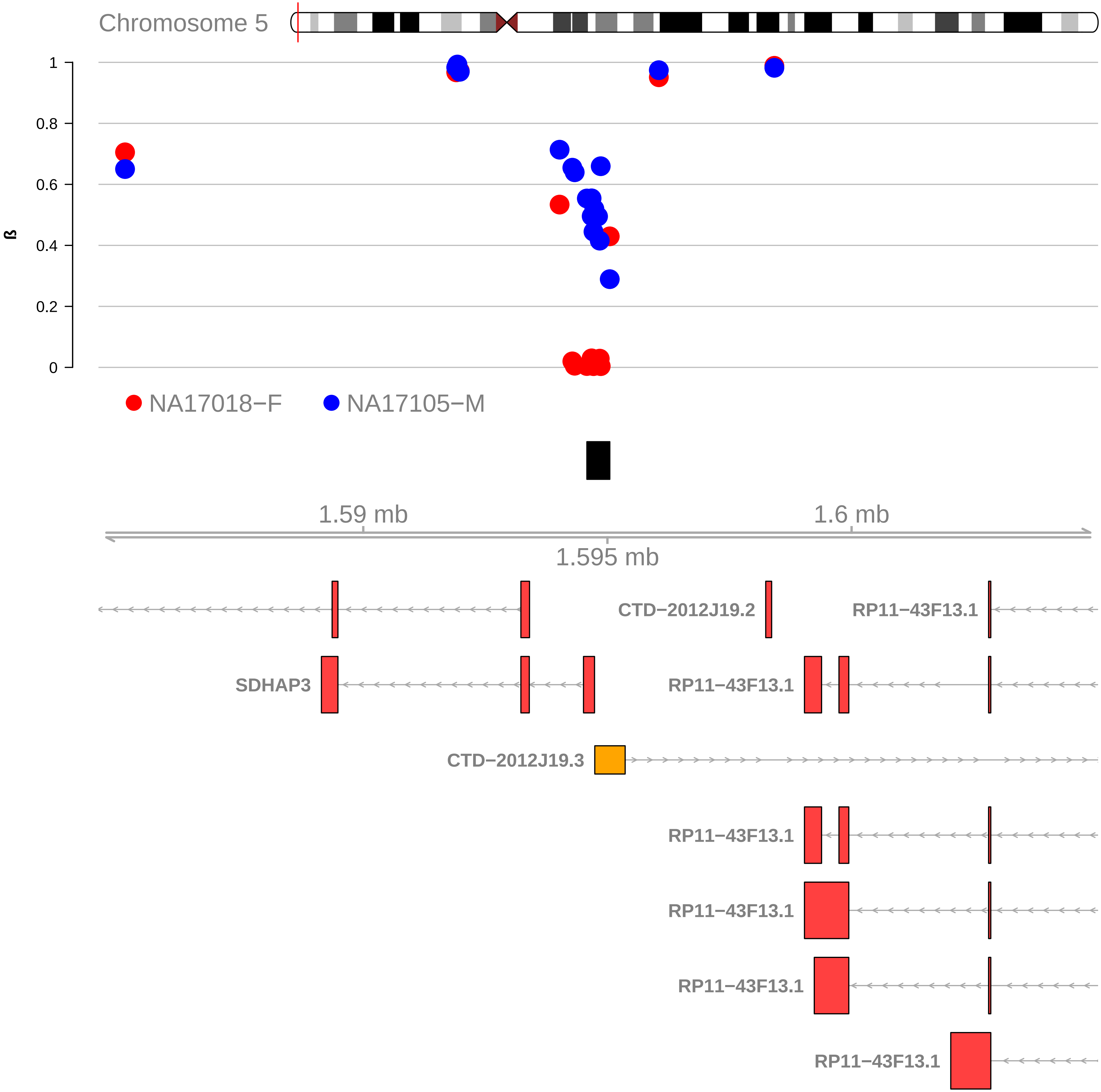
Chromosome 5
ß
1
0.8
0.6
0.4
0.2
0
NA17018−F
NA17105−M
1.59 mb
1.6 mb
1.595 mb
CTD−2012J19.2
RP11−43F13.1
SDHAP3
RP11−43F13.1
CTD−2012J19.3
RP11−43F13.1
RP11−43F13.1
RP11−43F13.1
RP11−43F13.1
